# Serum proteomics reveals a tolerant immune phenotype across multiple pathogen taxa in wild vampire bats

**DOI:** 10.3389/fimmu.2023.1281732

**Published:** 2023-12-12

**Authors:** Amanda Vicente-Santos, Lauren R. Lock, Meagan Allira, Kristin E. Dyer, Annalise Dunsmore, Weihong Tu, Dmitriy V. Volokhov, Claudia Herrera, Guang-Sheng Lei, Ryan F. Relich, Michael G. Janech, Alison M. Bland, Nancy B. Simmons, Daniel J. Becker

**Affiliations:** ^1^ School of Biological Sciences, University of Oklahoma, Norman, OK, United States; ^2^ Department of Tropical Medicine, School of Public Health and Tropical Medicine, Tulane University, New Orleans, LA, United States; ^3^ Vector-Borne and Infectious Disease Research Center, Tulane University, New Orleans, LA, United States; ^4^ Center for Biologics Evaluation and Research, U.S. Food and Drug Administration, Silver Spring, MD, United States; ^5^ Department of Pathology and Laboratory Medicine, School of Medicine, Indiana University, Indianapolis, IN, United States; ^6^ Hollings Marine Laboratory, Charleston, SC, United States; ^7^ Department of Biology, College of Charleston, Charleston, SC, United States; ^8^ Department of Mammalogy, Division of Vertebrate Zoology, American Museum of Natural History, New York, NY, United States

**Keywords:** *Desmodus rotundus*, bacteria, virus, protozoa, biomarker, shotgun proteomics

## Abstract

Bats carry many zoonotic pathogens without showing pronounced pathology, with a few exceptions. The underlying immune tolerance mechanisms in bats remain poorly understood, although information-rich omics tools hold promise for identifying a wide range of immune markers and their relationship with infection. To evaluate the generality of immune responses to infection, we assessed the differences and similarities in serum proteomes of wild vampire bats (*Desmodus rotundus*) across infection status with five taxonomically distinct pathogens: bacteria (*Bartonella* spp., hemoplasmas), protozoa (*Trypanosoma cruzi*), and DNA (herpesviruses) and RNA (alphacoronaviruses) viruses. From 19 bats sampled in 2019 in Belize, we evaluated the up- and downregulated immune responses of infected versus uninfected individuals for each pathogen. Using a high-quality genome annotation for vampire bats, we identified 586 serum proteins but found no evidence for differential abundance nor differences in composition between infected and uninfected bats. However, using receiver operating characteristic curves, we identified four to 48 candidate biomarkers of infection depending on the pathogen, including seven overlapping biomarkers (DSG2, PCBP1, MGAM, APOA4, DPEP1, GOT1, and IGFALS). Enrichment analysis of these proteins revealed that our viral pathogens, but not the bacteria or protozoa studied, were associated with upregulation of extracellular and cytoplasmatic secretory vesicles (indicative of viral replication) and downregulation of complement activation and coagulation cascades. Additionally, herpesvirus infection elicited a downregulation of leukocyte-mediated immunity and defense response but an upregulation of an inflammatory and humoral immune response. In contrast to our two viral infections, we found downregulation of lipid and cholesterol homeostasis and metabolism with *Bartonella* spp. infection, of platelet-dense and secretory granules with hemoplasma infection, and of blood coagulation pathways with *T. cruzi* infection. Despite the small sample size, our results suggest that vampire bats have a similar suite of immune mechanisms for viruses distinct from responses to the other pathogen taxa, and we identify potential biomarkers that can expand our understanding of pathogenesis of these infections in bats. By applying a proteomic approach to a multi-pathogen system in wild animals, our study provides a distinct framework that could be expanded across bat species to increase our understanding of how bats tolerate pathogens.

## Introduction

Bats are widely known to be reservoir hosts of various zoonotic pathogens, including viral [e.g., Nipah (1), Hendra (2), and Marburg (3)] and non-viral infections [e.g., *Bartonella* spp (4), *Trypanosoma* spp (5) and hemoplasmas (6)]. Interestingly, despite the presence of these pathogens, bats generally do not suffer from clinical disease, with a few documented exceptions [e.g., rabies virus (7)]. Although the immune mechanisms by which bats tolerate a wide range of infections remain unsolved, in part because immunological studies have been limited to a few bat species (8), an increasing body of evidence suggests that bat tolerance of infection is likely due to distinct aspects of immunity that evolved alongside their unique ability to fly among mammals and their exceptionally long lifespans (9–11). Here and throughout, our discussion of tolerance refers to that of infection [i.e (12)] rather than to immunological tolerance [i.e., mechanisms that limit response against self-antigens (13)].

Flight is highly metabolic demanding and generates harmful free-radical byproducts that damage biologically relevant molecules such as DNA (14). To reduce self-DNA–mediated immunopathology, bats have evolved DNA repair mechanisms (9) and dampened endogenous DNA-sensing pathways (15, 16). In addition, genome-wide comparisons across bat species have revealed unique gene losses in bats that downregulate inflammasome pathways in three main areas: the natural killer (NK) gene complex, epithelial defense receptors, and the interferon (IFN)-γ–induced pathway (9, 15, 17, 18). These findings suggest that bats developed mechanisms that suppress virus-induced inflammatory responses relative to human immunopathology (19–21). Moreover, the dampened inflammasome response of bats has shown to have minimal effects on viral replication (22), revealing adaptations to control viral infections. For instance, some bats maintain constitutive type I IFN responses, limiting virus propagation and responding rapidly to infections (23, 24).

Because bat tolerance to pathogens may result from adaptations to cope with flight-induced cellular stress, it has been hypothesized that bats can elicit defenses against intracellular pathogens more effectively than extracellular pathogens (25). However, most research on the bat immune response to intracellular pathogens has focused on RNA viruses, whereas DNA viruses remain poorly understood. Although sensing mechanisms for DNA viruses may be dampened in bats [PYHIN family (15) and STING (16)], bats must evolve new ways of detecting exogenous and endogenous DNA, as DNA viruses have been detected and isolated in many bat species (26, 27). Additionally, bat immune responses against other intracellular pathogens (e.g., many bacteria) and extracellular pathogens (e.g., many protozoa) are even less studied. Because these non-viral infections are common and highly prevalent in wild bats (28, 29), distinct mechanisms may potentially exist to cope with these infections.

Here, we aimed to assess differences and similarities in the immunology of wild bats infected and uninfected with five distinct types of pathogen taxa: RNA (coronavirus) and DNA (herpesvirus) viruses, bacteria (*Bartonella* spp. and hemotropic mycoplasmas), and protozoa (*Trypanosoma cruzi*). In addition to spanning multiple pathogen taxa (and thus potentially eliciting different immune responses), these pathogens are sufficiently common in bats to test for immunological differences (5, 30–34). To comprehensively characterize bat immune phenotypes, we used serum proteomics, which provides a unique perspective into the immune system of bats given the small sample volumes required and the profiling of proteins informative of cellular responses in blood as well as proximal organs and tissues (35, 36). Proteomic tools are also a promising method to study bat immunology more broadly, expanding from the few bat colonies, cell lines, and species-specific reagents currently available to conduct such research (8). However, proteomics has only been applied to a few bat systems (33, 36–38). We expanded a previous proteomic study of common vampire bats (*Desmodus rotundus*), an epidemiologically relevant species due to their tendency to feed on blood of livestock, wildlife, and occasionally humans (33, 39, 40). Vampire bats are infected by a wide variety of pathogens and, importantly, have a high-quality genome annotation available for protein identification (41, 42). We evaluated up- and down-regulated immune responses of infected and uninfected individuals within each pathogen and compared responses across pathogen taxa. Although we expected to find some general protein markers of infection, we predicted that bats would elicit differential immune responses according to the type of pathogen (i.e., DNA and RNA viruses, bacteria, or protozoa).

## Methods

### Vampire bat sampling

As described previously (33), and as part of long-term studies of vampire bat diet, immunity, and infection (43, 44), we captured 19 vampire bats in 2019 in the Lamanai Archeological Reserve in northern Belize using mist nests and a harp trap placed at a tree roost entrance. After recording morphometric data and sex, age, and reproductive status, we lanced the propatagial vein with a sterile 23G needle and collected blood into a serum separator tube (BD Microtainer) with a heparinized capillarity tube. We centrifuged the sample to separate serum from blood cells and inactivated the serum heating at 56°C for one hour before storing at -80°C until further analyses. We also collected whole blood on Whatman FTA cards as well as oral and rectal swabs in DNA/RNA Shield (Zymo), stored at –20°C and –80°C, respectively. We followed guidelines for the safe and humane handling of bats from the American Society of Mammalogists (Sikes & Gannon 2011), and our methods were approved by the Institutional Animal Care and Use Committee of the American Museum of Natural History (AMNHIACUC-20190129). Sample collection was authorized by the Belize Forest Department (permit FD/WL/1/19(09). Serum samples for proteomic analysis were approved by the National Institute of Standards and Technology Animal Care and Use Coordinator under approval MML-AR19-0018.

### Pathogen detection

We previously used RT-PCR to screen oral and rectal swabs from these bats for coronaviruses (targeting the RNA-dependent RNA polymerase [RdRp] gene), finding relatively moderate prevalence (4/19) of viruses in the genus *Alphacoronavirus* (33). Here, we used additional molecular tests to screen paired blood samples for *Trypanosoma cruzi*, hemotropic mycoplasmas (hereafter hemoplasmas), and *Bartonella* spp., as well as the same oral swabs for herpesviruses. These pathogens have been characterized in vampire bats specifically, for which they can obtain moderate-to-high prevalence (30, 45, 46). Following manufacturer protocols, we extracted DNA from blood on FTA cards using QIAamp DNA Investigator Kits (Qiagen) and total nucleic acids from oral swabs using a Quick-DNA/RNA Viral Kit (Zymo). Using previously published protocols, we used PCR to screen for *T. cruzi* (targeting two regions, the Satellite DNA (SatDNA) (47) and the minicircle kinetoplast DNA Miniexon gene [(kDNA; 48)], hemoplasmas [(targeting the 16S rRNA gene; 30)], *Bartonella* spp. [(nested PCR targeting the *gltA* gene; (45, 49)] and herpesviruses [nested PCR targeting the DNA polymerase gene (50)]. We used nuclease-free water as a negative control; for protozoan and hemoplasma PCRs, we included *T. cruzi* and *Candidatus* Mycoplasma haemozalophi as positive controls. PCR products were visualized by electrophoresis (1.5–3% agarose gel containing SYBR Safe (ThermoFisher Scientific) or GelRed (Biotum) nucleic acid gel stain. The *T. cruzi* primers used are highly specific and do not require sequencing confirmation, whereas all hemoplasma PCR–positive amplicons were submitted to Psomagen for sequence confirmation. To reduce risks of cross-contamination from nested PCRs, we did not include positive controls for *Bartonella* spp. and herpesviruses; instead, amplicons of expected size (approximately 300 bp and 200 bp, respectively) were submitted to the NCSU Genomic Sciences Laboratory for sequence confirmation.

### Protein digestion and proteomic profiling

We processed bat serum samples using the S-Trap method for digestion with the S-Trap micro column (ProtiFi, ≤ 100 μg binding capacity), as described in full detail previously (33). Briefly, 2 μL of serum (≈ 100 μg protein) was reduced with DL-Dithiothreitol (DTT) and alkylated with 2-chloroacetamide (CAA). Proteins were digested with trypsin (1:30 mass ratio), followed by a one-hour incubation at 47°C. After reducing the resulting peptides to dryness in a vacuum centrifuge at low heat, we reconstituted the samples with 100 μL 0.1% formic acid before being analyzed using an UltiMate 3000 Nano LC coupled to a Fusion Lumos Orbitrap mass spectrometer (ThermoFisher Scientific). After using the trap elute setup PepMap 100 C18 trap column (ThermoFisher Scientific), peptides were separated on an Acclaim PepMap RSLC 2 µm C18 column (ThermoFisher Scientific) at 40°C. Full details on the LC-MS/MS method applied and the data-independent acquisition (DIA) settings used are provided previously (33).

As described before (33), we used the DIA-NN software suite and used the NCBI RefSeq *Desmodus rotundus* Release 100 GCF_002940915.1_ASM294091v2 FASTA (29,845 sequences) to search the vampire bat samples. We mapped identified bat proteins to human orthologs using BLAST+ (51) and custom python scripts (36) for downstream analyses using human-centric databases (33). When human orthologs did not exist, we used *ad hoc* ortholog identifiers.

### Data analysis

Our analyses included 19 samples and a pooled serum sample as a quality control. The digestion was evaluated by the number of peptide spectral matches. Considering the limitations of working with a small sample size from wild animals, we established conservative cut-offs for data interpretation. For proteomic data analyses, we imputed missing abundance values by estimating half of the minimum observed intensity of each protein (52). Missing values were excluded for descriptive presentation of means and log_2_-fold change (LFC).

For pathogen infection analyses, we used principal component analysis (PCA) to reduce the dimensionality of our identified protein dataset, using abundances scaled and centered to have unit variance. For each of our five pathogen types (including coronaviruses), we used a permutation multivariate analysis of variance (PERMANOVA) with the *vegan* R package (53). To identify differentially abundant proteins between uninfected and infected bats, we used a two-sided Wilcoxon rank sum, using the Benjamini–Hochberg (BH) correction to adjust for the inflated false discovery rate (54). Next, we calculated the LFC as the difference in mean log_2_-transformed abundance between uninfected and infected bats. To identify candidate biomarkers of infection, we used receiver operating characteristic (ROC) curve analysis using the *pROC* package (55). We generated the area under the ROC curve (AuROC) to measure classifier performance and estimated their 95% confidence intervals (CI) with the *pROC* package using 2000 stratified bootstrap replicates. We considered proteins with AuROC ≥ 0.9 to be strict classifiers of pathogen positivity, whereas proteins with AuROC ≥ 0.8 but less than 0.9 were considered less conservative (56). Only proteins with a lower CI bound above 0.5 were classified as strict or less conservative classifiers, as an AuROC of 0.5 suggests no discrimination (56). All other proteins were treated as poor classifiers (57). To visualize the matrix of candidate serum biomarkers, we used the *pheatmap* package with scaled and centered log_2_-transformed protein abundances and Ward’s hierarchical clustering method (58, 59).

Finally, we evaluated and compared up- and down-regulated responses to all pathogen infections using gene ontology (GO) and enrichment analysis. The *gprofiler2* package was used as the interface to the g:Profiler tool g:GOSt (60, 61). Our enrichment analysis was limited to candidate protein biomarkers with an AuROC ≥ 0.8 (i.e., less conservative candidates). We determined the up- and down-regulated proteins using LCF. Using the AuROC value, we ranked proteins and performed incremental enrichment testing, with the resulting p-values adjusted by the Set Counts and Sizes (SCS) correction. We restricted our data sources to GO biological process (BP), cellular component (CC), molecular function (MF), the Kyoto Encyclopedia of Genes and Genomes (KEGG), and WikiPathways (WP). None of the eight bat proteins lacking human orthologs were candidate biomarkers (AuROC < 0.8). Hence, manual GO and pathway mapping were not required.

## Results

**Figure 1:**
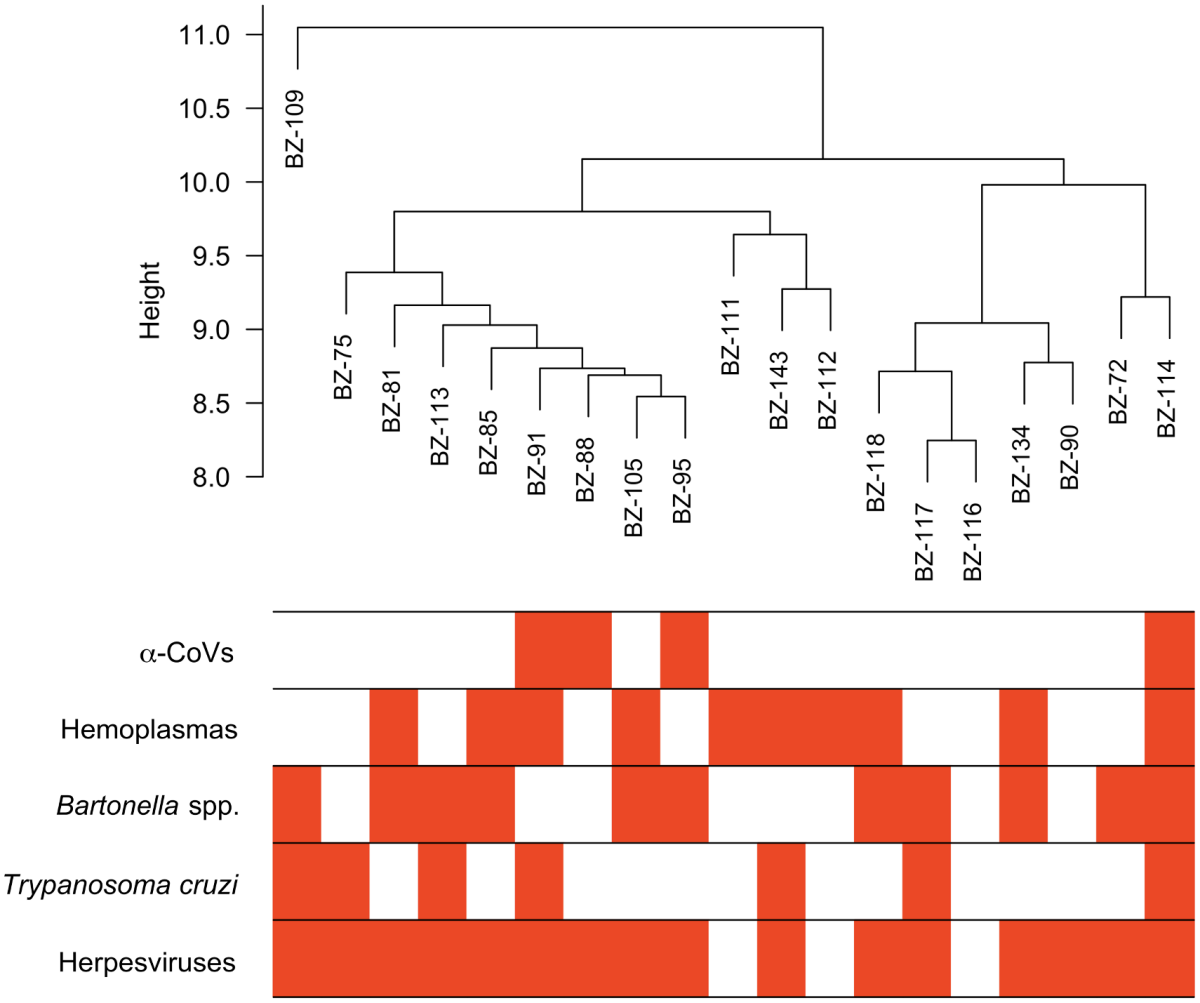
Hierarchical cluster of the 19 vampire bat samples based on their serum proteome and infection heatmap showing which individuals are PCR-positive (red) for the five selected pathogens.

From our previous study (33), we identified 586 proteins in these vampire bat serum samples using bottom-up proteomics with DIA. Four of the 19 bats sampled were positive for α-CoVs, 16 for herpesviruses, ten for hemoplasmas, 11 for *Bartonella* spp., and seven for *Trypanosoma cruzi*. Most bats were infected by at least one pathogen (95%), with 79% of bats having two or more infections; one bat was negative for all pathogens, and another bat was co-infected by all pathogens (**Figure 1**).

### Contrasting proteomic profiles with pathogen infection

**Figure 2:**
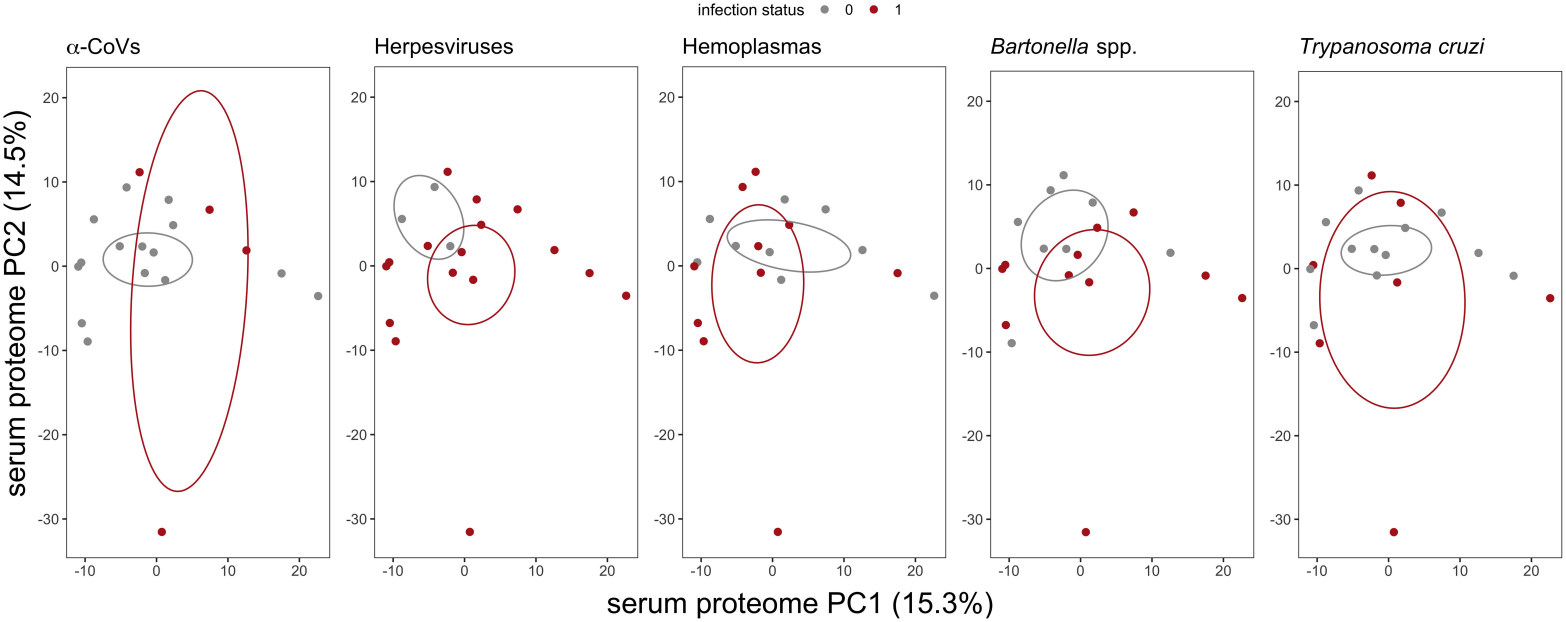
Biplot of the first two principal components (PCs) from the PCA of the 586 identified serum proteins in vampire bats. Individual bats are colored by their infection status, where red represents PCR-positive for any of the five selected pathogens. Ellipses display the standard error of infected or uninfected group centroids using the *ggordiplot* package. Missing abundance values were imputed as half the minimum intensity per protein.

**Figure 3:**
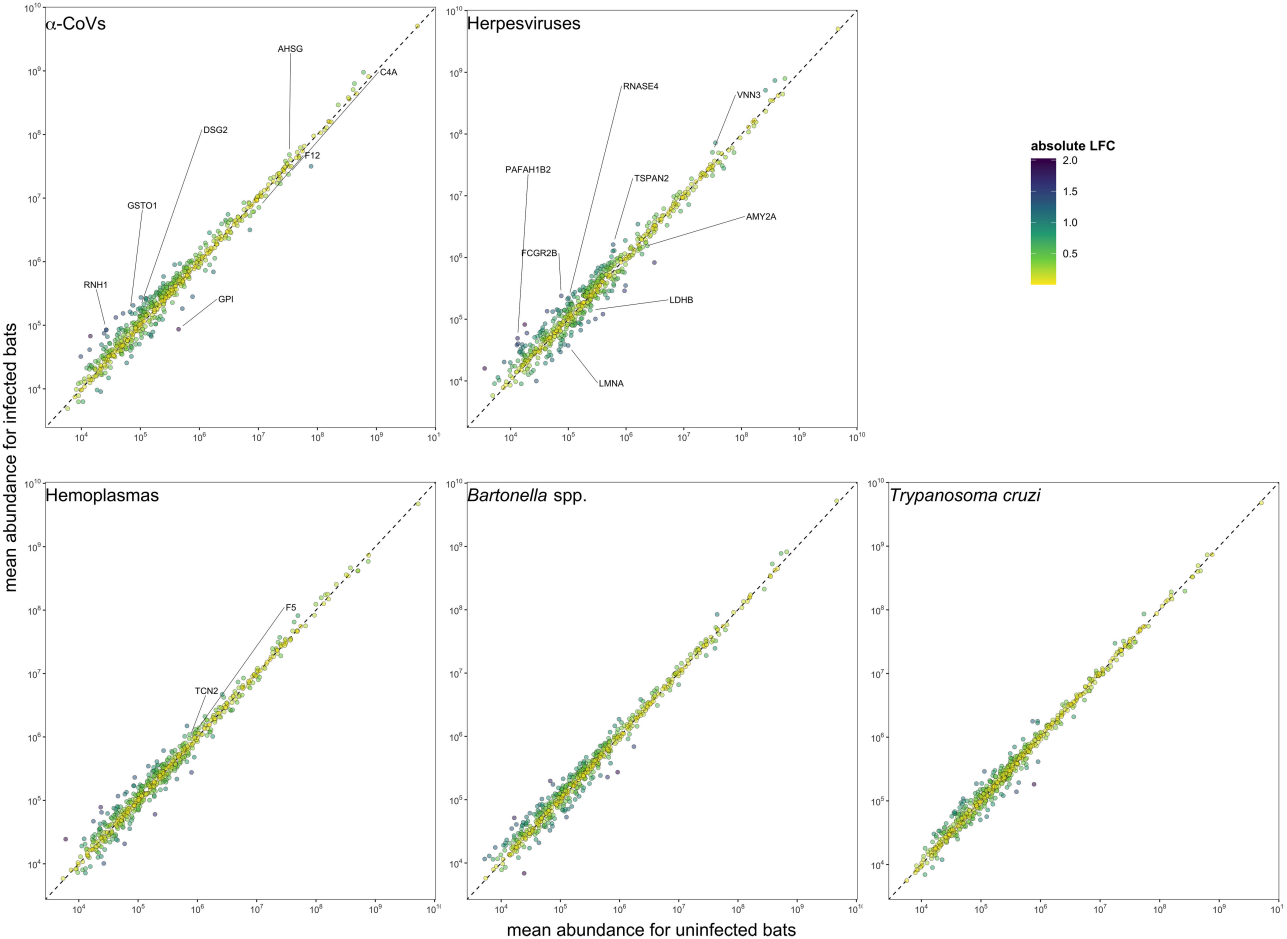
Mean protein abundance across the 586 identified proteins for uninfected and infected bats for the five selected pathogens. The dashed lines show the 1:1 reference for comparison. Strict candidate biomarkers (AuROC ≥ 0.9) were only found in α-CoVs (n=7), herpesviruses (n=8), and hemoplasmas (n=2), and are labeled with gene symbols (**Table 2**). Missing values were excluded prior to determining mean abundances.

We used multivariate tests to evaluate differences in serum proteomes between infected and uninfected bats for each of our five pathogen types. The first two principal components (PCs) explained 29.8% of the variance of the plasma proteome (**Figure 2**). For all pathogens, we found no difference in proteome composition by infection status (α-CoVs: F_1,17_ = 0.348, R^2^ = 0.020, p = 0.767; herpesviruses: F_1,17_ = 1.167, R^2^ = 0.064, p = 0.290; hemoplasmas: F_1,17_ = 1.513, R^2^ = 0.082, p = 0.211; *Bartonella* spp.: F_1,17_ = 1.730, R^2^ = 0.092, p = 0.168; and *T. cruzi*: F_1,17_ = 0.395, R^2^ = 0.023, p = 0.720). Similarly, Wilcoxon rank sum tests (BH adjusted p < 0.05) found no proteins with significant differential abundance between infected and uninfected bats for any pathogen (**Figure 3**).

**Figure 4:**
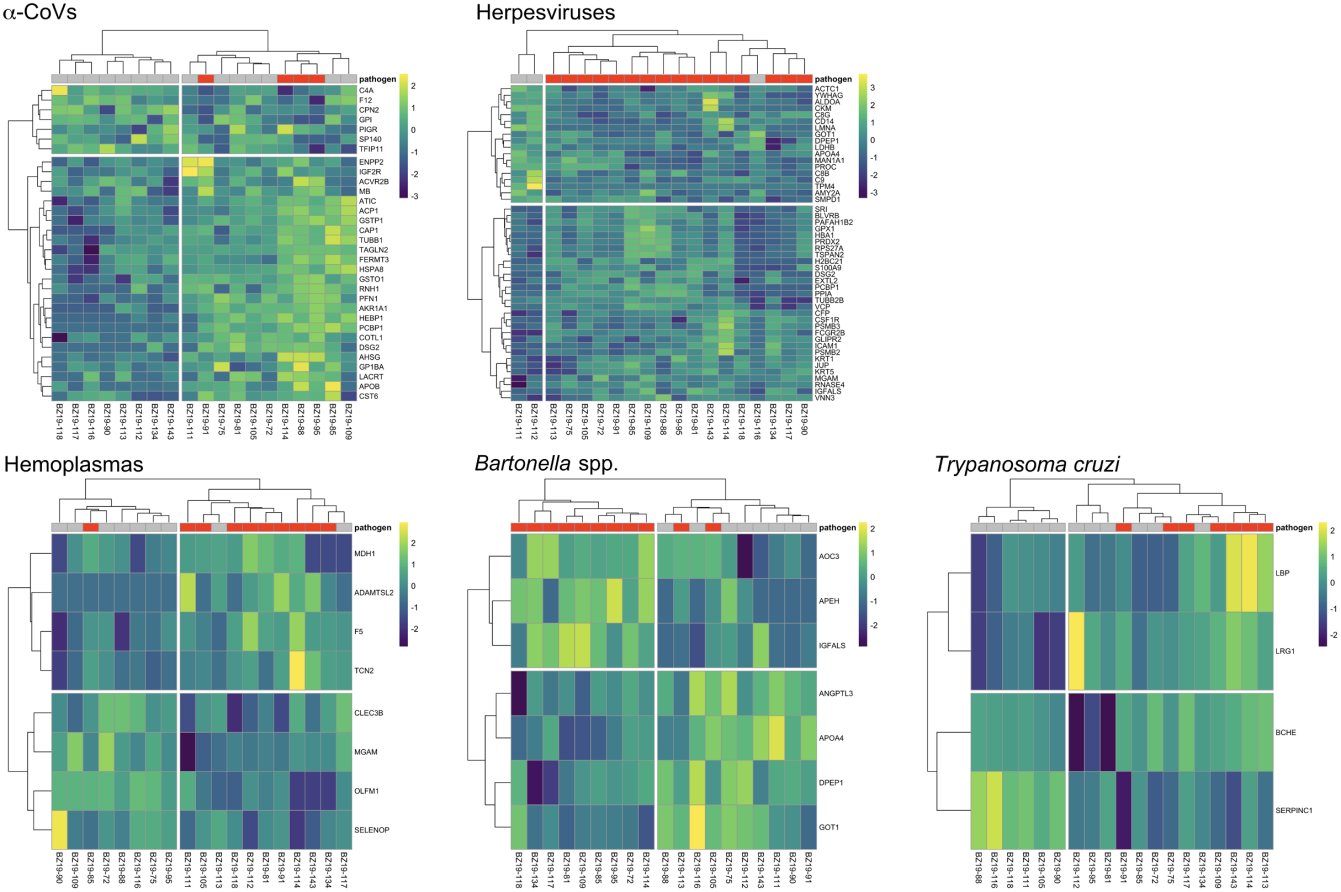
Heatmaps of log_2_-transformed abundance scaled to a mean of zero for candidate serum biomarkers (AuROC ≥ 0.8) for α-CoV (n=32), herpesvirus (n=48), hemoplasma (n=8), *Bartonella* spp. (n=7) and *T. cruzi* (n=4) infections. Columns display individual bats and rows represent proteins as gene symbols. Infection status is indicated at the top of the heatmap, where red represents PCR-positive status for any given pathogen. Ward’s hierarchical method was used for clustering (58).

However, using less-conservative classifier cutoffs (AuROC ≥ 0.8), ROC curve analyses identified 92 candidate proteins across pathogens, ranging from four for *T. cruzi* to 48 for herpesviruses, and provided strong discriminatory power to differentiate between proteomic profiles of uninfected and infected bats (**Figure 4**). In addition, seven putative biomarkers of herpesvirus infection overlapped with putative biomarkers of all other pathogens except *T. cruzi* (**Table 1**; **Figure S1**). All shared putative protein biomarkers tended to predict infection in the same direction for both pathogens, except MGAM, which was elevated in herpesvirus infections but reduced in hemoplasma infections (**Table 1**). When using stricter classifier cutoffs (AuROC ≥ 0.9), we only identified seven putative biomarkers for α-CoV infection, eight for herpesviruses, two for hemoplasmas, and none for *T. cruzi* or *Bartonella* spp. (**Figure 3**; **Table 2**; **Figure S2**).

**Table 1:** Shared candidate serum biomarkers (AuROC ≥ 0.8) between two pathogen infections.

UniProt Gene Name	Protein description	Pathogen	LFC	AuROC	95% CI
DSG2	desmoglein-2-like	Herpesviruses	0.831	0.812	0.690-0.935
		α-CoVs	0.921	0.900	0.753-1.000
PCBP1	poly(rC)-binding protein 1	Herpesviruses	1.048	0.812	0.690-0.935
		α-CoVs	1.173	0.867	0.698-1.000
MGAM	maltase-glucoamylase, intestinal	Herpesviruses	0.534	0.833	0.611-1.000
		Hemoplasmas	-0.423	0.833	0.632-1.000
APOA4	apolipoprotein A-IV	Herpesviruses	-0.558	0.833	0.614-1.000
		*Bartonella* spp.	-0.618	0.898	0.748-1.000
DPEP1	Dipeptidase 1	Herpesviruses	-1.703	0.896	0.677-1.000
		*Bartonella* spp.	-1.461	0.852	0.657-1.000
GOT1	aspartate aminotransferase, cytoplasmic	Herpesviruses	-0.754	0.833	0.586-1.000
		*Bartonella* spp.	-0.602	0.812	0.593-1.000
IGFALS	insulin-like growth factor-binding protein complex acid labile subunit	Herpesviruses	0.526	0.854	0.656-1.000
		*Bartonella* spp.	0.440	0.807	0.596-1.000

The log_2_-fold change (LFC) and AuROC with its associated 95% confidence interval (CI) of each of the seven proteins are given per pathogen.

**Table 2:** Strict candidate serum biomarkers (AuROC ≥ 0.9) found for α-CoV, herpesvirus, and hemoplasma infections.

Pathogen	UniProt Gene Name	Protein description	LFC	AuROC	95% CI
α-CoVs	AHSG	α-2-HS-glycoprotein isoform X1	0.525	0.908	0.719-1.000
	C4A	complement C4-A-like	-0.455	0.967	0.888-1.000
	DSG2	desmoglein-2-like	0.921	0.900	0.753-1.000
	F12	coagulation factor XII	-0.512	0.933	0.814-1.000
	GPI	glucose-6-phosphate isomerase	-2.358	0.958	0.875-1.000
	GSTO1	glutathione S-transferase ω-1	1.288	0.900	0.746-1.000
	RNH1	ribonuclease inhibitor	1.682	0.967	0.888-1.000
Herpesviruses	AMY2A	pancreatic α-amylase	-0.397	0.938	0.815-1.000
	FCGR2B	low affinity immunoglobulin γ Fc region receptor II-b	1.664	0.979	0.921-1.000
	LDHB	L-lactate dehydrogenase B chain	-0.782	0.917	0.773-1.000
	LMNA	lamin	-1.402	0.917	0.784-1.000
	PAFAH1B2	platelet-activating factor acetylhydrolase IB subunit β isoform X1	1.898	0.917	0.778-1.000
	RNASE4	ribonuclease 4	1.132	0.938	0.798-1.000
	TSPAN2	tetraspanin-2	1.438	0.938	0.824-1.000
	VNN3	vascular non-inflammatory molecule 3-like	0.688	0.917	0.778-1.000
Hemoplasmas	F5	coagulation factor V	0.346	0.922	0.806-1.000
	TCN2	transcobalamin-2	0.371	0.911	0.784-1.000

The log_2_-fold change (LFC) and AuROC with its associated 95% confidence interval (CI) are given for each protein.

### Enrichment analyses reveal distinct pathways across pathogens

**Figure 5:**
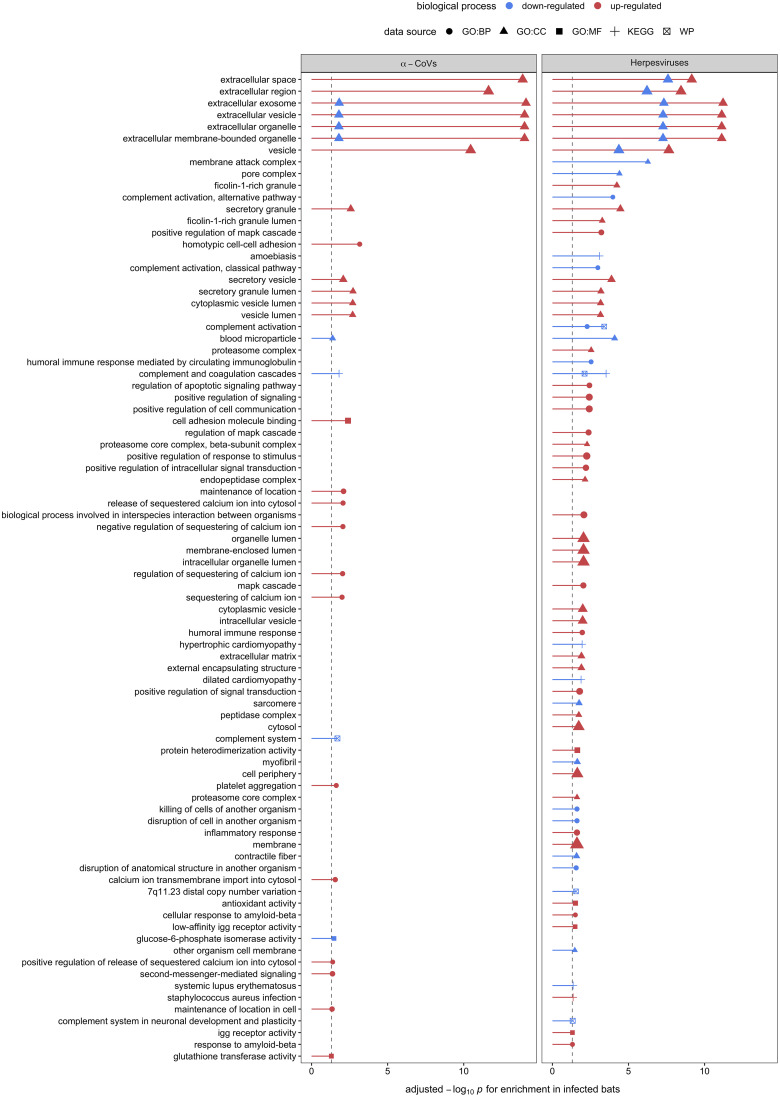
Enrichment analyses of the 32 and 48 candidate serum biomarkers of α-CoV and herpesvirus infections, respectively. Up- (red) and downregulation (blue) of biological processes are displayed. Processes are labeled by source: gene ontology (GO) biological process (BP), cellular component (CC), and molecular function (MF), the Kyoto Encyclopedia of Genes and Genomes (KEGG), and WikiPathways (WP).

**Figure 6:**
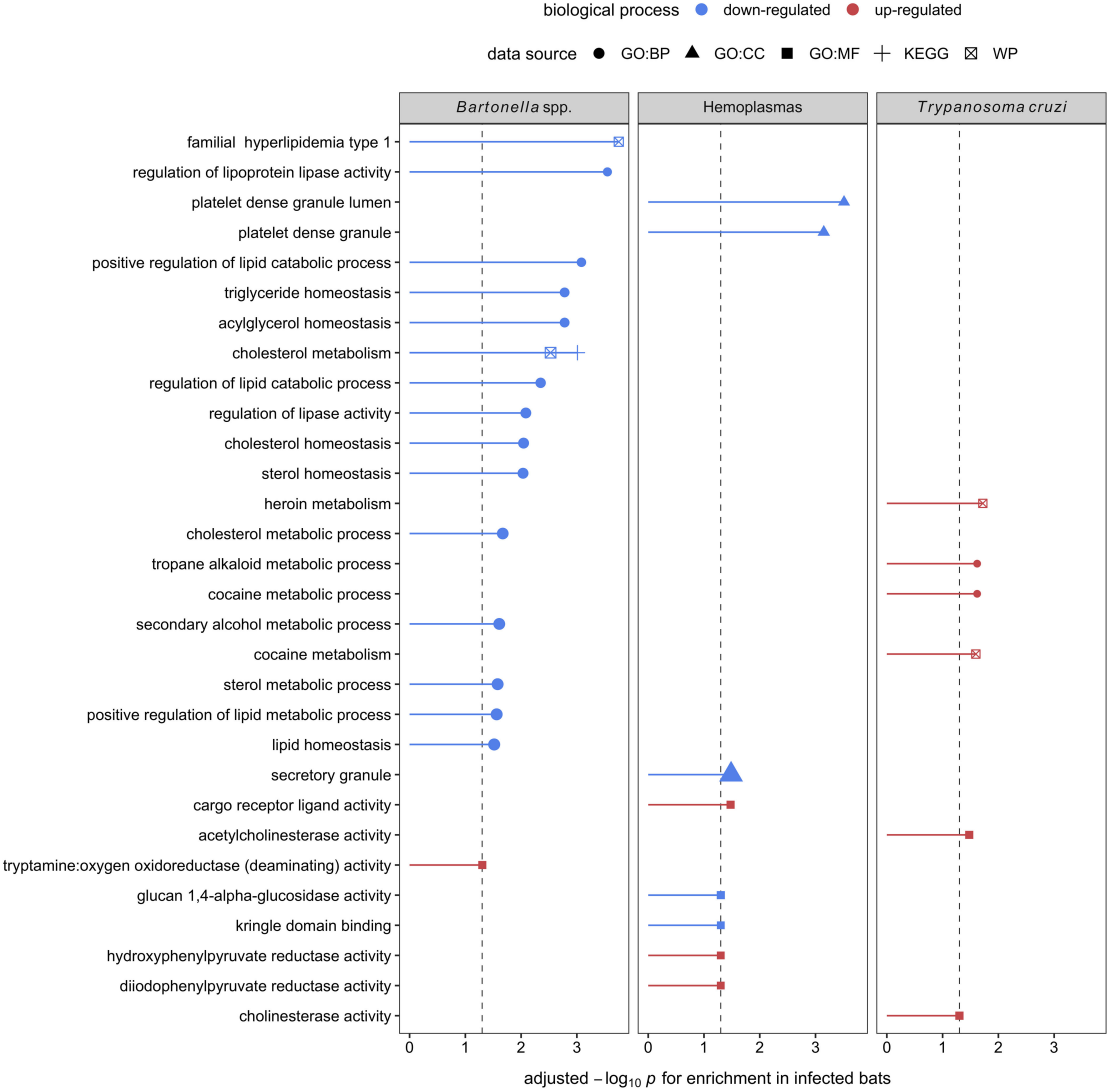
Enrichment analyses of the seven, eight, and four candidate serum biomarkers of *Bartonella* spp., hemoplasma, and *T. cruzi* infections, respectively. Up- (red) and downregulation (blue) of biological processes are displayed. Processes are labeled by source: gene ontology (GO) biological process (BP), cellular component (CC), and molecular function (MF), the Kyoto Encyclopedia of Genes and Genomes (KEGG), and WikiPathways (WP).

Using GO terms, we assessed and compared up- and down-regulated responses to each pathogen type. Enrichment analyses of the less-conservative putative biomarkers (AuROC ≥ 0.8) revealed multiple functional proteomic differences between infected and uninfected bats and across pathogen taxa. Infected bats with our viral pathogens, but not our bacterial or protozoan pathogens, had upregulation of extracellular and cytoplasmatic secretory vesicles and downregulation of complement activation and coagulation cascades (**Figure 5**). Moreover, herpesvirus infections elicited downregulation of leukocyte-mediated immunity but upregulation of MAPK cascades and the inflammatory and humoral response (**Figure 5**). In contrast to our two viral infections, we found downregulation of platelet-dense and secretory granules with hemoplasma infections, lipid and cholesterol homeostasis as well as metabolism with *Bartonella* spp. infection, and blood coagulation pathways with *T. cruzi* (**Figure 6**). Interestingly, *T. cruzi* infections also elicited upregulation of neurotransmitter pathways, including choline and drug metabolic processes, such as cocaine, heroin, and alcohol (**Figure 6**).

## Discussion

In recent decades, there has been an increasing interest in understanding bat immune responses to infections, particularly those involving viruses (17, 62, 63). However, insights into how bat immune systems respond to other pathogen taxa (e.g., bacteria and protozoa) are also relevant given their high infection prevalence and zoonotic potential (25, 28). Proteomic tools have provided valuable insights into how bats cope with infections, especially in wild populations (33, 36–38). Here, we assessed the differences and similarities in serum proteomes of wild vampire bats infected and uninfected with five divergent pathogen taxa: RNA and DNA viruses (α-CoVs and herpesviruses, respectively), bacteria (hemoplasmas and *Bartonella* spp.), and protozoa (*T. cruzi*). By evaluating potential protein biomarkers of infection as well as the up-and down-regulated physiological responses of infected vampire bats, our approach identified interesting differences and similarities across pathogen taxa.

Although none of our five pathogens were significantly associated with serum protein composition nor abundance, we identified 17 (strict) to 92 (less-conservative) candidate protein biomarkers across pathogen taxa using ROC curve analyses. From these 92 biomarkers, DSG2 and PCBP1 were shared positive predictors, with both proteins elevated in bats infected with α-CoVs or herpesviruses. Both proteins are involved in the mediation of cell–cell adhesion and innate antiviral responses. PCBP1 is produced by activated T cells to stabilize the innate immune response (64, 65) via mitochondrial antiviral signaling and prevents virus-related inflammation (66), whereas DSG2 is a known receptor of adenovirus in humans (67) that, in response to pro-inflammatory cytokines, induces epithelial cells to apoptosis (68). We also identified shared biomarkers between herpesvirus and bacterial (*Bartonella* spp.) infections; APOA4, DPEP1, and GOT1 were negative predictors while IGFALS was a positive predictor. The decreased abundance of APOA4, DPEP1, and GOT1 shows downregulation of a pro-inflammatory response. In humans, APOA4 is upregulated in severe adenovirus community-acquired pneumonia (69), and plasma concentrations of the protein increase in inflammatory disorders during hepatitis B virus infections (70). However, COVID-19 patients also show downregulation of the apolipoprotein, possibly associated with macrophage function (71). DPEP1, conversely, is associated with neutrophil-mediated inflammatory responses, facilitating neutrophil recruitment from to bloodstream to inflamed tissue by acting as a physical adhesion receptor (72). GOT1 plays a role in T cell exhaustion, as it maintains chronic immune responses by regulating CD8+ effector and memory T cell generation (73, 74). In contrast, upregulation of IGFALS shows activation of the innate immune response. In mice, IGFALS is virus-inducible and is essential in antiviral responses by enhancing interferon production (75).

Both herpesvirus and hemoplasma infections also shared MGAM as a putative biomarker, but this protein was a positive predictor for these viruses and a negative predictor for these bacteria. MGAM is involved in breaking down carbohydrates in the small intestine, and its deficiency has been linked to gastrointestinal diseases (76, 77). Although MGAM seems to have a role in neutrophil biology (78) and may have a role in regulating inflammatory responses in the gastrointestinal tract, which contains the largest population of mast cells in the body (79), little is known about its immune function, and our results highlight a potential distinction between viral and bacterial infections.

When focusing on strict biomarkers in this analysis, we confirmed the same seven proteins for α-CoVs as in our prior study (33) and added eight (positive biomarkers: FCGR2B, PAFAH1B2, RNASE4, TSPAN2, and VNN3; negative biomarkers: AMY2A, LDHB, and LMNA) for herpesvirus infections and two (F5 and TCN2, both positive) for hemoplasma infections. Vampire bats likely respond to herpesvirus infections in multiple ways, possibly due to the ability of these viruses to persist as latent infections that can reactivate periodically (80). By increasing abundance of FCGR2B, bats are apparently mounting humoral immune responses against herpesviruses, as this protein is expressed in mature neutrophils in charge of removing spontaneously forming immune complexes to dampen Fc-dependent immune reactions (81). This mechanism may possibly arise to prevent the potentially toxic side effects of systemic innate interferon (IFN) responses, especially important for chronic infections or secondary exposure to the virus (82). However, the up-regulation of PAFAH1B2, which induces large amounts of platelets to aggregation, may instead indicate acute viral infections, as it has been documented in patients with avian influenza (H1N9) acute-phase infections (83) that show high levels of inflammatory cytokines and induce vascular permeability (84, 85). Moreover, RNases, such as RNASE4, are acutely induced during infection and downregulated during prolonged infection periods (86). The increased abundance of RNASE4 also shows that bats present immunoprotective responses that directly degrade viral RNA to prevent viral replication or prompt host cell apoptosis (87). Cell-mediated responses also likely play a major role in herpesvirus infections, as we observed upregulation of both TSPAN2, a transmembrane protein expressed in neutrophils that modulates the inflammatory response, cell migration, and differentiation (88), and VNN3, an ectoenzyme secreted by neutrophils that is involved in oxidative stress and inflammation (89, 90). Finally, the downregulation of LDHB and LMNA may further indicate active viral replication. Decreased abundance of LDHB has been reported in human HIV infections, and the reduced abundance of this protein is hypothesized to give virions a higher probability of survival and intercellular transmission (91). Nuclear lamins, such as LMNA, normally impede viral infectivity and replication (92), and viruses, such as herpes simplex virus, induce lamin alterations (93).

By contrast, the strict biomarkers we identified for hemoplasma infections suggest these pathogens only activate cellular pathways. To impede bacterial dissemination, bats upregulate F5, a central regulator of hemostasis involved in clotting and macrophage and neutrophil reclusion (94). This protein is also involved in forming neutrophil extracellular traps, which intensify an inflammatory response (95, 96). On the other hand, the role of the upregulation of TCN2, a protein involved in vitamin B12 metabolism, is not clear (97). However, a recent study shows that B12 levels are significantly decreased in patients with tuberculosis infections (98), underlying a potential avenue of future research in bacterial infections.

Enrichment analysis on the 32 and 48 putative biomarkers from α-CoV– and herpesvirus-infected bats displayed strong upregulation of extracellular and cytoplasmatic secretory vesicles and downregulation of complement activation and coagulation cascades. These observations further indicate active viral replication, as the exosome and other extracellular vesicles are known to be exploited by both DNA and RNA to facilitate spread among cells (99–101). These mechanisms can also mediate intercellular communication during innate and adaptive immune responses. The secretion of vesicles from leucocytes with immune modulatory properties, such as NK cytotoxicity, T cell activation and proliferation, and the ability of cells to produce IFN-γ, or delivering innate immune effectors (e.g., interleukins), are well-documented host responses to viral infections that these specific viruses could also hijack to evade the same responses [reviewed in (102)]. Unfortunately, without antibody-based depletion, our analyses could only characterize the most abundant proteins found in serum, limiting our ability to detect low-abundant proteins such as cytokines that could give us a better understanding of host–virus interactions (103).

Interestingly, herpesvirus infections, but not α-CoV infections, also elicited downregulation of leukocyte-mediated immunity and upregulation of mitogen-activated protein kinase (MAPK) cascades and the inflammatory and humoral response. Besides the particularity of herpesviruses to persist in the host as latent infections (80), studies suggest that bat responses to DNA viruses are dampened in contrast to RNA viruses (15, 16, 104), potentially explaining our observed patterns. DNA viruses are known to usurp the MAPK signaling pathway to exploit DNA replication machinery, induce cell proliferation, and prevent cell death in response to pathogen recognition (105). Herpesviruses, particularly, have evolved their ability to manipulate the host’s cell mechanisms to achieve enhanced permissiveness and endure latent infections, including the MAPK pathways (106). In addition, the upregulation of the MAPK pathway by viruses seems to be involved with the downregulation of IFN production and the consequent impairment of the cellular innate immune response, benefiting viral replication (107, 108). Our results here suggest that bats also respond to DNA viruses by increasing their inflammatory and humoral response.

In contrast, enrichment analyses on the eight putative biomarkers of hemoplasma infection showed downregulation of platelet-dense and secretory granules. Platelets play a crucial role in the innate immune system by being the first responders to an injury and directing the immune response to any pathogen that may be present due to the injury (109). It is unclear, however, if their downregulation here indicates a bacterial evasion mechanism or if it is elicited by the host to limit pro-inflammatory damage. For *Bartonella* spp., the seven candidate biomarkers indicated downregulated lipid metabolic processes. Although, to our knowledge, there are no studies specifically evaluating host–*Bartonella* spp. interactions in lipid metabolism, studies of another intracellular bacteria, *Mycoplasma* spp., show that bacterial infections can manipulate host lipid metabolic processes in many ways (110–113). Lipids play important roles in pathogen docking, invasion, and intracellular trafficking as well as membrane synthesis during pathogen replication and persistence (114). Therefore, mycoplasmas have developed mechanisms sequestering host cell lipids, such as cholesterol, to promote survival (112). These include inhibiting the degradation of bacteria in lysosomes as well as using host lipids as an energy source and providing the building blocks for pathogen assembly (112). Simultaneously, the downregulation of lipid metabolism (sterol) modulates host immune responses to mycobacterial infections mediated by IFN-γ (113, 115). Thus, evidence shows that the downregulation of mechanisms involved in lipid metabolism could act as a double-edged sword favoring the persistence of bacterial infections (113). Interestingly, lipid metabolic processes were not altered for hemoplasmas, suggesting high variance in the bat immune response to intracellular bacteria.

Finally, enrichment analysis on the four identified biomarkers of *T. cruzi* infections showed downregulation of blood coagulation and upregulation of neurotransmitter pathways, including choline and drug metabolic processes. Downregulation of blood coagulation pathways coincides with *T. cruzi* infections in humans and mice showing anemia, leukocytosis (i.e., increased white blood cell counts), and thrombocytopenia (i.e., low count of platelets in blood), especially in acute infection phases (116–120). On the other hand, while the upregulation of neurotransmitter pathways is not described in human or mice *T. cruzi* infections, another trypanosome (*T. brucei*, which causes sleeping sickness) does show neurological manipulations of the host (121, 122). This is an intriguing finding that reveals a potential avenue for future research.

Overall, our findings show that a multi-pathogen approach can reveal a similar suite of immune mechanisms responding to select viral infections that is distinct from those to other studied pathogen taxa. These distinct immune responses to viral and non-viral infections are consistent with classic and recent findings in vertebrates (123, 124) as well as in other bat species (125). Further, we identify potential biomarkers that could expand our understanding of pathogenesis in bats. However, we recognize that our small sample size may limit the precision and generality of our findings, and larger sample sizes moving forward will allow similar studies to more robustly identify biomarkers through cross-validation approaches (126). Moreover, working with free-ranging animals presents intrinsic limitations. While in experimental laboratory settings, researchers can often use a few model animals by minimizing heterogeneity in their genetic structure and treatments (127), this is virtually impossible with wild animals. Likewise, even as we screened for five divergent and common pathogens in vampire bats, these animals could also be infected by other pathogens not considered in our study, possibly confounding results. Additionally, while the timing of infections and co-infections is known to influence immune response [e.g (128, 129)], such data are typically not available for wildlife. Despite these limitations, we show that information-rich approaches such as proteomics show promise in interrogating the bat immune response in the wild. This is particularly important for future studies, as our understanding of bat immunology has been restricted to a few species from which we have bat colonies, cell lines, or species-specific reagents (8). The field of bat immunology will advance greatly when expanding proteomics approaches to controlled experimental settings and comparing these responses across multiple bat species and diverse ecological settings.

## Data availability statement

As reported previously (33), the method file (85min_DIA_40x21mz.meth) and mass spectrometry proteomics data are available in the ProteomeXchange Consortium via the PRIDE partner repository with identifier PXD031075.

## Ethics statement

The animal study was approved by Institutional Animal Care and Use Committee of the American Museum of Natural History. The study was conducted in accordance with the local legislation and institutional requirements.

## Author contributions

AV: Conceptualization, Formal Analysis, Investigation, Methodology, Visualization, Writing – original draft, Writing – review & editing. LL: Data curation, Investigation, Methodology, Writing – review & editing. MA: Data curation, Investigation, Methodology, Writing – review & editing. KD: Data curation, Investigation, Methodology, Writing – review & editing. AD: Data curation, Investigation, Methodology, Writing – review & editing. WT: Data curation, Investigation, Methodology, Writing – review & editing. DV: Data curation, Investigation, Methodology, Writing – review & editing. CH: Data curation, Investigation, Methodology, Writing – review & editing. GL: Data curation, Investigation, Methodology, Writing – review & editing. RR: Data curation, Investigation, Methodology, Writing – review & editing. MJ: Data curation, Investigation, Methodology, Writing – review & editing. AB: Data curation, Investigation, Methodology, Writing – review & editing. NS: Data curation, Funding acquisition, Investigation, Methodology, Resources, Writing – review & editing. DB: Conceptualization, Data curation, Formal Analysis, Funding acquisition, Investigation, Methodology, Project administration, Resources, Supervision, Writing – review & editing.
