# Supplementary material for: Serum proteomics reveals a tolerant immune phenotype across multiple pathogen taxa in wild vampire bats
Source: Front Immunol. 2023 Dec 12;14:1281732. doi: 10.3389/fimmu.2023.1281732 (PMC10773587; doi:10.3389/fimmu.2023.1281732)
Supplement: Supplementary file 1 [file DataSheet_1.docx]

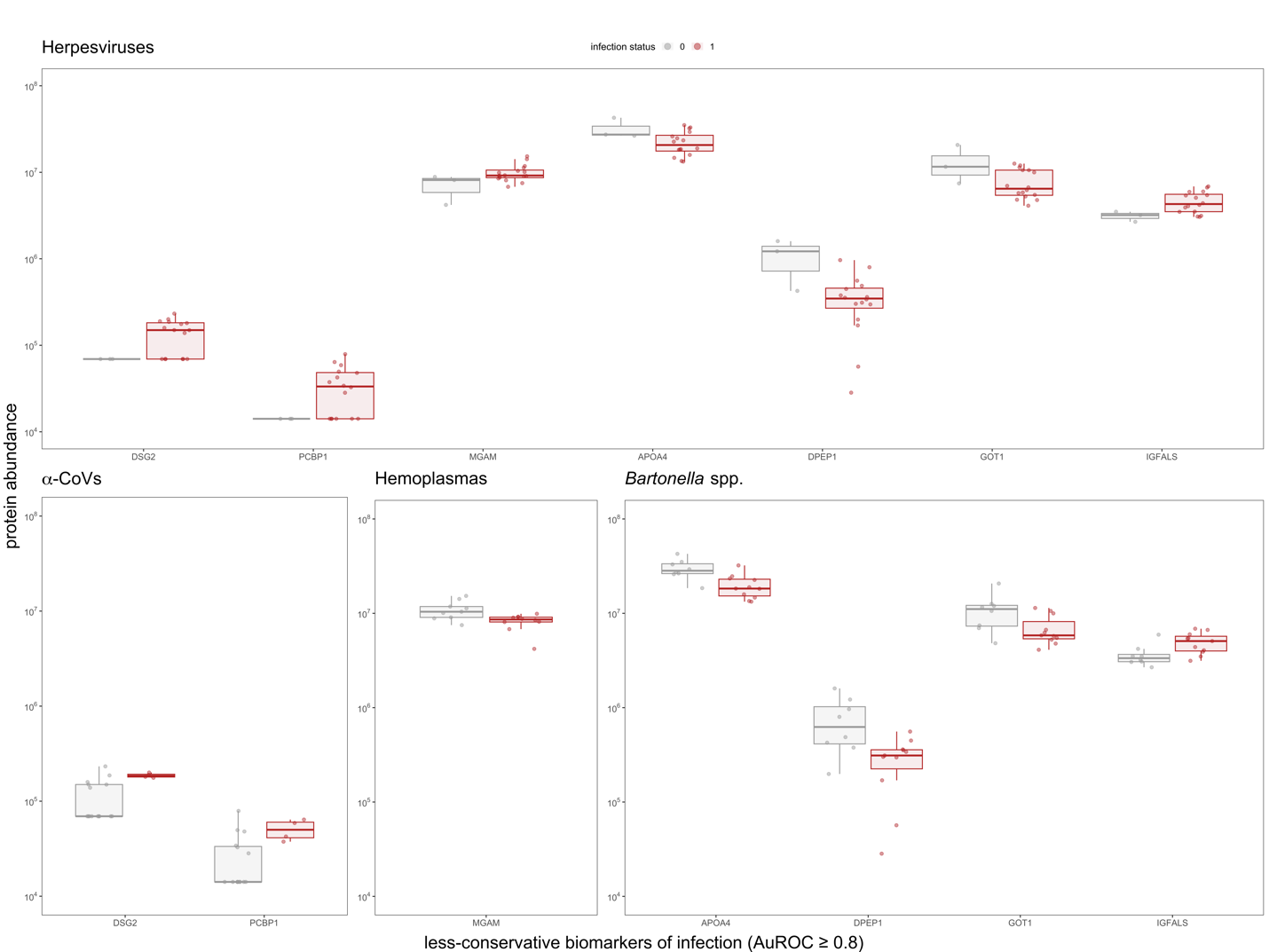


Figure S1. ﻿Protein abundance of the seven shared putative biomarkers of pathogen infection. All shared biomarkers were less conservative (0.8 ≤ AuROC > 0.9). Infected and uninfected vampire bats for each pathogen are shown in red and gray, respectively. Boxplots are overlaid by raw data jittered to reduce overlap.


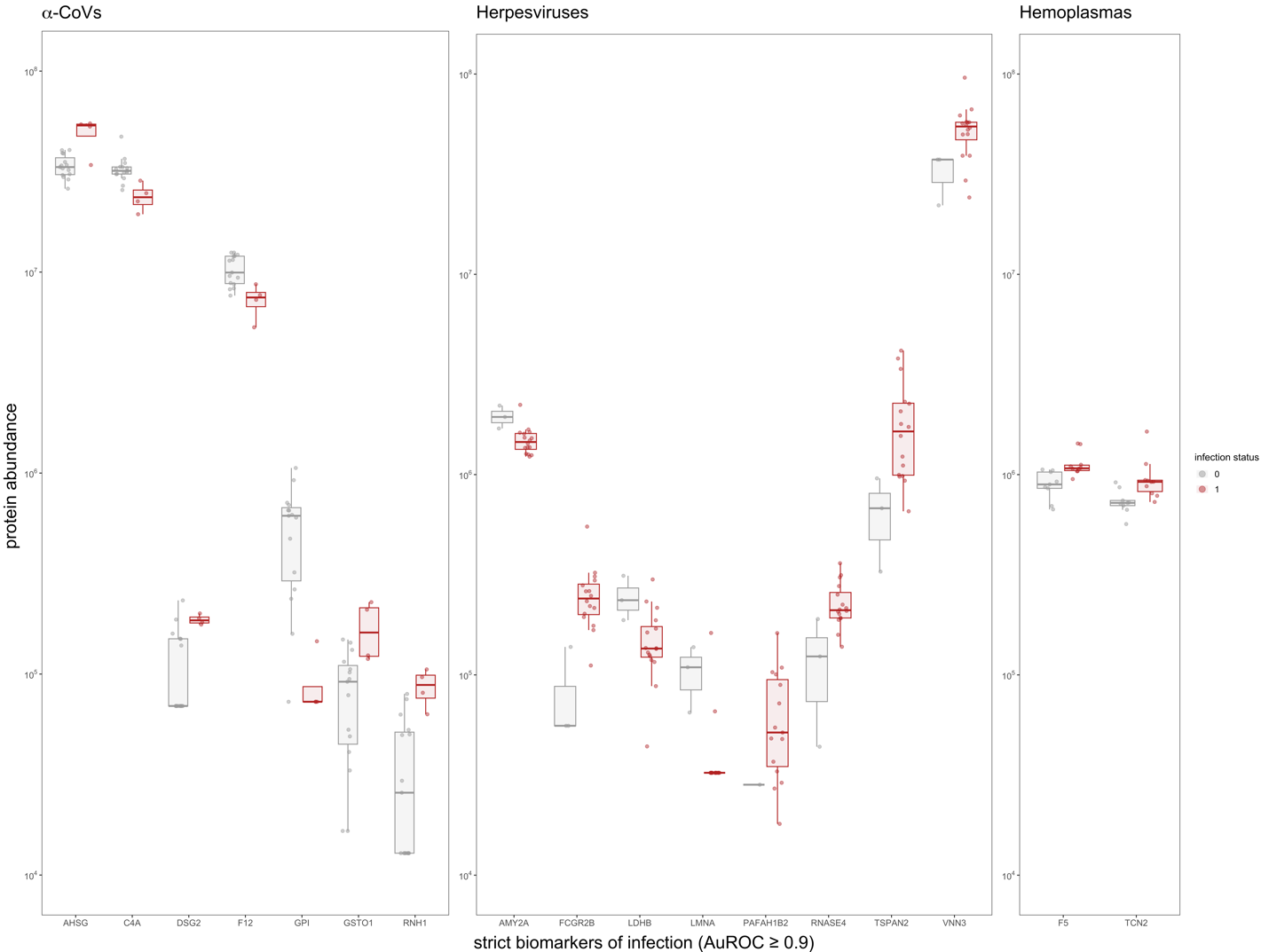


Figure S2. ﻿Protein abundance between infected (red) and uninfected (gray) vampire bats of the strict potential biomarkers of infection (AuROC ≥ 0.9) for α-CoVs, herpesviruses, and hemoplasmas. Boxplots are overlaid by raw data jittered to reduce overlap.
